# Inhalable Nanomaterial Discoveries for Lung Cancer Therapy: A Review

**DOI:** 10.3390/pharmaceutics17080996

**Published:** 2025-07-31

**Authors:** Iqra Safdar, Syed Mahmood, Muhammad Kumayl Abdulwahab, Suzita Mohd Noor, Yi Ge, Zarif Mohamed Sofian

**Affiliations:** 1 Department of Pharmaceutical Technology, Faculty of Pharmacy, Universiti Malaya, Kuala Lumpur 50603, Malaysia; safdariqra155@gmail.com (I.S.); syedmahmood@um.edu.my (S.M.); 2 Universiti Malaya-Research Centre for Biopharmaceuticals and Advanced Therapeutics (UBAT), Department of Pharmacology, Faculty of Medicine, Universiti Malaya, Kuala Lumpur 50603, Malaysia; 3 Faculty of Pharmaceutical Sciences, Chulalongkorn University, PathumWan, Bangkok 10330, Thailand; 4 Centre of Advanced Materials (CAM), Faculty of Engineering, Universiti Malaya, Kuala Lumpur 50603, Malaysia; 5 Department of Chemistry, Faculty of Science, Universiti Malaya, Kuala Lumpur 50603, Malaysia; kumaylabdul@um.edu.my; 6 Department of Biomedical, Faculty of Medicine, Universiti Malaya, Kuala Lumpur 50603, Malaysia; suzita@um.edu.my; 7 School of Pharmacy, Queen’s University Belfast, Belfast BT9 7BL, UK

**Keywords:** nanomedicine, inhalable nanomaterials, inhalable devices, lung cancer, targeted drug delivery

## Abstract

Lung cancer remains one of the most common and deadliest forms of cancer worldwide despite notable advancements in its management. Conventional treatments, such as chemotherapy, often have limitations in effectively targeting cancer cells, which frequently lead to off-target side effects. In this context, the pulmonary delivery of inhalable nanomaterials offers the advantages of being rapid, efficient, and target-specific, with minimal systemic side effects. This concise review summarizes the basic research and clinical translation of inhalable nanomaterials for the treatment of lung cancer. We also provide insights into the latest advances in pulmonary drug delivery systems, focusing on various types of pulmonary devices and nanomaterials. Furthermore, this paper discusses significant challenges in translating the discoveries of inhalable nanomaterials into clinical care for lung cancer and shares strategies to overcome these issues.

## 1. Introduction

Cancer is a complex disease driven by genetic and epigenetic changes, influenced by environmental factors and the microbiome, and it remains the second leading cause of death worldwide [1]. Among the various types of cancer, lung cancer stands out as the primary cause of cancer-related deaths globally, with an annual death toll of 1.76 million and a high incidence rate [2]. Its complexity arises from a combination of non-genetic, epigenetic, and genetic variables, which complicate its diagnosis and treatment [3,4]. Non-small cell lung cancer (NSCLC) and small cell lung cancer (SCLC) are the two main subtypes of lung cancer, with NSCLC accounting for the majority of cases and posing challenges due to a poor prognosis and a late diagnosis. Cigarette smoking is a major contributor to lung cancer deaths [5]. Despite advancements in oncology, non-small cell lung cancer (NSCLC) often evades early detection and has a low overall 5-year survival rate. Conventional treatments for lung cancer include surgery, chemotherapy, and radiotherapy, with chemotherapy playing a significant role [6]. Although some adjuvant therapies have been introduced in recent years to enhance the effectiveness of lung cancer treatment, these strategies have not significantly improved overall patient survival. Suboptimal drug delivery efficiency, drug resistance, and severe systemic side effects remain among the most common challenges. underscores the urgent need for more effective approaches to treating lung cancer [7]. Oral and parenteral routes remain the preferred methods for administering chemotherapeutics like cisplatin and carboplatin in lung cancer treatment, typically resulting in better distribution of these cytotoxic drugs after administration, often leading to significant off-target toxicity [8]. However, in light of the limitations associated with systemic drug administration, alternative strategies are being explored—most notably,

Pulmonary drug delivery is preferred for respiratory diseases because it provides localized action with minimal systemic effects [9]. This preference is supported by the lung’s surface area, thin alveolar blood barriers, relatively low enzymatic activity, and rich vascularization, which allow inhaled drugs to achieve higher concentrations in the lungs and enable easier administration [10]. The extracellular matrix (ECM) is a dynamic and organized network of macromolecules that provides mechanical support and biochemical signals to surrounding cells. In healthy lungs, the ECM is essential for maintaining tissue architecture, facilitating gas exchange, and regulating processes such as cell adhesion, migration, and repair. It consists of fibrillar collagens (types I, III, IV), elastin, adhesive glycoproteins (fibronectin, laminin, vitronectin), and proteoglycans (decorin, biglycan, perlecan), along with glycosaminoglycans like hyaluronan [11].

While the ECM preserves tissue structure and elasticity in normal lungs, its composition and mechanical properties undergo significant alterations in lung cancer [12]. ECM stiffness increases dramatically, with macro metastases exhibiting a tenfold increase compared to healthy lung tissue [12]. The ECM critically regulates the behavior of nanoparticles NPs in biological systems by modulating their penetration, distribution, and therapeutic efficacy. In the tumor and lung microenvironments [13]. The surface chemistry of NPs influences their interactions with ECM components like fibronectin, potentially disrupting normal ECM function [10]. When inhaled, NPs with different functional groups exhibit varying intrapulmonary distributions and inflammatory responses, which correlate with their protein corona composition. Understanding these interactions is crucial for improving nanomaterial design and clinical translation in cancer treatment [13].

Additionally, this knowledge aids in assessing health risks associated with inhaled ultrafine particles and informs the development of more effective nanoparticle-based strategies for tumor targeting and diagnosis [10]. Nano-based systems provide additional diagnostic benefits during lung cancer treatment, including imaging, screening, and monitoring [10]. NPs offer promising avenues for drug delivery to both the bloodstream and lungs, with each route presenting unique challenges and opportunities. The interaction between NPs and biological environments differs significantly between the bloodstream and the lung extracellular matrix (ECM). In the lungs, pulmonary surfactant protein A (SP-A) enhances NP uptake by alveolar macrophages, while albumin in plasma decreases it [14]. In the bloodstream, proteins adsorb onto NPs, forming a protein corona (PC) that influences their behavior and fate [15]. The protein corona formed on NPs in lung surfactant is distinct from that in plasma, with a prevalence of lipid-binding proteins, such as surfactant protein A (SP-A) and surfactant protein D (SP-D) [16]. Interestingly, the lipid composition of the corona remains relatively consistent across different NP types in lung surfactants [16]. Inhaled NP therapies offer advantages such as targeted lung delivery, enhanced stability, and reduced systemic side effects [17]. Despite its benefits, pulmonary delivery is still in its early stages of research, and several issues, such as pharmacology, immunology, and toxicity, must be considered for the development of acceptable inhalable nano-based chemotherapeutic agents [18]. To address the current challenges in inhalable therapies, significant research has focused on nanoparticle-based delivery systems, which offer versatile platforms for enhancing pulmonary drug targeting and therapeutic outcomes.

Nanoparticles (NPs) have emerged as promising drug delivery systems, offering targeted and efficient delivery of therapeutics. Various types of nanocarriers are being studied, including polymeric nanoparticles, liposomes, dendrimers, micelles, and microparticles [19]. Compared to macro-sized particles, NPs possess a greater surface-to-volume ratio. This allows for higher drug loading capacity and the ability to form multiple interactions with various molecules, including receptors on the surface of target cells. By leveraging the enhanced permeability and retention (EPR) effect, NPs promote targeted drug accumulation at the site of interest, reducing exposure to healthy tissues and minimizing adverse side effects while improving bioavailability [20].

This review aims to provide a comprehensive assessment of the use of drug delivery systems based on inhalable nanoparticles for the targeted treatment of lung cancer. It also discusses an overview of lung cancer, including its prevalence and established treatment modalities. Subsequently, the review scrutinizes the characteristics of various nanoparticles employed in inhalable liposomes, with polymeric nanoparticles serving as examples of drug delivery mechanisms, emphasizing their capacity to enhance treatment efficacy. The review examines targeting strategies, encompassing both passive and active mechanisms, and surveys the diverse range of therapeutic agents administered via inhalable nanoparticles, spanning from chemotherapy to immunotherapy.

Additionally, the review addresses challenges inherent in this approach, such as potential pulmonary toxicity and the imperative for scalability in clinical applications. It also contemplates future research trajectories, such as personalized medicine and combination therapies. To summarize, this review provides an invaluable resource for researchers and clinicians seeking to gain a comprehensive understanding of the current state and prospects of drug delivery by inhalable nanoparticles in the treatment of targeted lung cancer.

## 2. Delivery Devices for Pulmonary Drug Delivery

**Figure 1:**
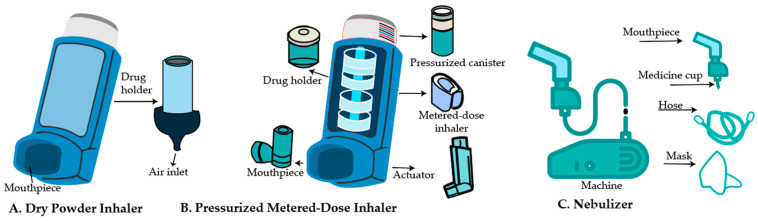
Schematic illustration of clinically used inhalable devices for pulmonary drug delivery. The figure depicts the three main classes of inhalation devices used in clinical practice: (**A**) Dry Powder Inhaler (DPI)—a breath-actuated device delivering drug powder to the lungs; (**B**) Pressurized Metered Dose Inhaler (pMDI)—a propellant-based system that releases a fixed dose of aerosolized medication; and (**C**) Nebulizer—a device that converts liquid formulations into inhalable aerosols.

The two determining factors for successful inhalation therapy are the optimal pharmacological properties of NPs and the delivery devices. Dry powder inhalers (DPIs), pressurized metered dosage inhalers (pMDIs), and nebulizers are the three primary inhalation devices used to provide drugs to the lungs [21] (Figure 1).

### 2.1. Selection Criteria for Inhalation Devices Based on Nanomaterial Properties in Lung Cancer Therapy

In addition to designing nanocarriers for inhalation drug delivery, selecting a suitable aerosol device, such as nebulizers and inhalers (including soft mist, pressurized metered-dose, and dry powder), is also a critical consideration [22]. Historically, these devices have been evaluated for delivering nanocarrier-based drugs [22]. Pressurized metered-dose inhalers (pMDIs) deliver nanocarriers by mixing them with a propellant that releases a precise drug dose upon actuation [23]. Breath-actuated devices, such as dry powder inhalers, are well-suited for delivering moisture-sensitive formulations, including genes or gene-nanocarrier complexes, especially after they have undergone drying techniques like lyophilization, spray drying, or aerosolization to achieve the required aerodynamic size [24]. The delivery of nanocarriers to deeper lung regions can be effectively accomplished using propellant-free aerosols, commonly referred to as soft mist inhalers [25]. Metered-dose inhalers are limited by their inability to deliver large aerosol volumes and require precise patient coordination, which can hinder effective drug delivery to targeted lung cancer sites [26]. Nebulizers are widely used for the pulmonary delivery of nanocarriers due to their ability to produce aerosols with sufficiently large droplet sizes, enabling prolonged administration and effective deposition in deep-seated tumor regions. Clinically, they have demonstrated an ability to generate fine particle fractions conducive to optimal lung deposition at relatively low drug doses. Nonetheless, the therapeutic efficiency of nebulized formulations is influenced by critical physicochemical parameters, including surface tension, osmolarity, active pharmaceutical ingredient concentration, nebulization rate, and aerosol output flow rate [27]. In summary, selecting an appropriate inhalation device tailored to the physicochemical properties of the nanocarrier and the therapeutic target Table 1 within the lungs is crucial for maximizing drug delivery efficiency and clinical outcomes in lung cancer treatment.

### 2.2. Dry Powder Inhalers (DPIs)

DPIs are used to deliver high-dose medications directly to the lungs, offering promise in the treatment of various pulmonary and systemic diseases [29]. These devices can be made with micronized particles or lactose-based mixtures to enhance medication delivery to the lungs and potentially throughout the body [30]. They provide several benefits in pulmonary drug delivery, including direct deposition in the deep lungs and the potential for systemic drug administration [31]. Research on dry powders for inhalation has investigated both carrier-based mixtures and lactose-free formulations [32]. Traditional DPI formulations use micronized drugs mixed with coarse carriers, but their efficacy is low (12–30% lung delivery). Micronization can create amorphous domains, which can affect performance. Nano and micro-composite particles produced through wet milling and spray drying present a promising alternative [33]. Several factors influence the performance of DPIs, including device design, particle size, flow properties, formulation, drug-carrier adhesion, and respiratory flow rate [34], device design, particularly the piercing aperture location and inlet (Figure 1). The flow rate has a significant impact on device emptying and particle deposition [29]. DPI devices are currently classified into three major types based on their design: first-generation, single-dose DPIs; second-generation, multiple-dose DPIs; and third-generation, commonly known as ‘active’ or power-assisted DPIs. The first generation, such as the Rotahaler^®^ (GlaxoSmithKline, Brentford, London, UK) and the newer Handihaler^®^ (Boehringer Ingelheim, Ingelheim, Germany) and Breezhaler^®^ (Novartis Pharma, Basel, Switzerland), consists of breath-activated, single-dose devices in which a capsule of powder is perforated in the device with needles fixed to pressure buttons. With these inhalers, drug delivery is influenced by particle size and de-agglomeration of drug carrier agglomerates or mixtures delivered by the patient’s inspiratory flow. Some newly developed DPIs or existing devices are designed for new powder formulations. Proper usage of the inhaler is vital for achieving the intended therapeutic benefits [35]. Challenges in DPI development include optimizing powder dispersion and understanding drug-device-patient interactions to enhance lung deposition [36]. There is a wide selection of breath-activated or power-driven DPI devices on the market that can deliver one or several doses. Nonetheless, since a device’s design influences its performance, new and innovative device designs are still being developed. Combining suitable powder compositions with DPI designs that generate small-particle aerosols remains a challenge [37].

The powder formulation is aerosolized through a DPI device, where drug particles are separated from the carrier (from drug carrier mixtures or de-agglomerated drug particles), and the dose is delivered into the patient’s deep lungs. In these systems, particle size and flow properties, formulation, drug-carrier adhesion, respiratory flow rate, and the design of DPI devices significantly affect performance [34]. The airflow resistance of the DPI design impacts lung deposition, varying from 0.02 to 0.2 cm H_2_O/L/min. Low-resistance DPIs require a flow rate exceeding 90 L/min [38]. Second-generation DPIs encompass multidose devices that measure doses from a reservoir and multi-unit devices that dispense pre-metered doses. Examples include the Turbuhaler^®^ and Diskus, which represent a distinct category [35]. Second-generation DPIs, such as NEXThaler, Ellipta, and Genuair, provide convenient multidose delivery with specific drug combinations for asthma and COPD [39]. Aclidinium bromide, when delivered via inhaler, achieves high lung deposition in healthy subjects, with approximately 30% of the metered dose reaching the lungs [40,41].

### 2.3. Pressurized Metered-Dose Inhalers (pMDIs)

The most widely used drug delivery devices for inhalation therapy are nebulizers, pressurized metered-dose inhalers (pMDIs), and dry powder inhalers (DPIs), with pMDIs being the most favored and the most frequently prescribed by both physicians and patients [42]. Riker Laboratories introduced the pressurized metered-dose inhaler (pMDI) in 1956, marking a pivotal advancement in pharmaceutical aerosol delivery [43]. that revolutionized the industry. Despite significant technological progress, including modern inhaler systems with improved delivery efficiency, ensuring proper patient use remains a challenge, as highlighted by a Japanese study, which found that 24.2% of patients misused pMDIs [44,45]. The effectiveness of pMDIs can be attributed to their key components: an aluminum canister, a metering valve, and an actuator (Figure 1) [46]. These devices use propellants such as “HFA 134a” or “HFA 227” to produce a short burst of aerosol that delivers precise, pre-measured amounts of medication straight to the lungs [47]. The aerodynamic behavior of these NPs is crucial for effective delivery, with factors such as particle size, morphology, and density influencing their deposition in the airways [47]. Their performance is greatly influenced by the aerodynamic particle size distribution [46].

Moreover, the propellants used in pMDIs, such as hydrofluoroalkanes (HFAs), have been found to affect oxygen levels in valved holding chambers and ventilator delivery devices [48]. In terms of formulation, suspension formulations in pMDIs often incorporate surfactants to prevent bubble coalescence, resulting in a more stable discharge. Additionally, the metering valve plays a critical role in ensuring precise dosing, making the uniformity of dose mass and dose of tiny particles, which are crucial characteristics [49,50]. Formulation variables, such as surfactant and cosolvent concentrations, also significantly impact pMDI performance and lung deposition. For example, oleic acid, a surfactant, can increase lung deposition of beclomethasone dipropionate solution from 24% to 46%, while ethanol, a cosolvent, may decrease lung deposition in suspension formulations [51].

### 2.4. Nebulizer

Nebulizers generate aerosols containing active substances, with jet, ultrasonic, and membrane types, each offering distinct advantages and limitations [52]. Nebulizers consist of a mask, a hose with a mouthpiece, and a medicine cup attached to a machine (Figure 1) that converts liquid medication into a mist for inhalation via the mouthpiece. Jet nebulizers, commonly used in clinical practice, produce aerosols via high-velocity gas impact, requiring a flow rate of 6–8 L/min and a fill capacity of 4–5 mL. Despite their widespread use, these nebulizers have notable limitations, including significant drug losses and dependency on patient capacity [53]. In contrast, vibrating mesh nebulizers are more efficient and less affected by humidification, making them preferable in specific settings [54]. Ultrasonic nebulizers, which utilize ultrasonic vibrations to generate aerosol droplets, enable control over droplet size through adjustment of the oscillation frequency [55]. However, while effective in delivering inhaled pharmaceutical aerosols, nebulizers often suffer from poor lung delivery efficiency, leading to medication wastage and prolonged delivery times [56]. The evolution of nebulizer technology, particularly with the advent of vibrating mesh nebulizers, has significantly enhanced the therapeutic potential of these devices [57]. Further improvements in nubilizer efficacy can be achived through secondry devices and stratgies such as those that modifiey aerosol size distribution, synchronize drug adminstration with inhalation, reduces system deposionational loses, enhance the pacient interface and guide pacient breathing [56]. Studies have investigated the operating parameters of vibrating mesh nebulizers, finding that droplet size decreases with increasing resonance frequency [58]. These studies have also hightlited the advantages of vibrating mesh nublizer, including consistant drug delivery and low shear force [59].

Additionally, the effect of electrolyte concentration on droplet size and output rate in vibrating membrane nebulizers advances our understanding of their performance and prospective applications. Optimization of ultrasonic nebulizers can be achieved by using a single-frequency ultrasonic atomizer, capable of generating uniform atomized droplets [60]. Droplet size can be further controlled by tuning actuation parameters [60]. A high-frequency vibration system for liquid atomization has also been developed, producing highly dispersed aerosols with specific droplet sizes [61].

Despite their advantages, ultrasonic nebulizers are limited by their inability to nebulize suspensions and the potential to heat the liquid drug, rendering them unsuitable for thermolabile medications. However, they operate silently and efficiently nebulize solutions [62]. The latest advancements in nebulizer technology, particularly with vibrating mesh nebulizers like the eFlow^®^ and MicroAir^®^, have overcome the drawbacks of Traditional Jet and ultrasonic nebulizers. These modern nebulizers offer advantages such as reduced drug loss, noiseless operation, and enhanced portability [63]. They are also capable of aerosolizing a wide range of pharmaceutical formulations without compromising their integrity [58]. Notably, the new generation of nebulizers utilizing hybrid resonant acoustic (HYDRA) technology has demonstrated excellent aerosol deposition performance in vivo [64]. These technological breakthroughs show considerable promise. For improving drug delivery and patient adherence [65].

### 2.5. Other Inhaler Technology

The Respimat^®^ Soft Mist Inhaler is a portable device developed to improve medicine delivery to the lungs while reducing patient coordination requirements and improving ease of use [66]. The new reusable Respimat inhaler maintains performance and ensures consistent drug delivery, unaffected by the design [67]. Additionally, it also boasts environmental benefits by reducing the environmental impact compared to traditional Pressurized, metered-dose inhalers [63]. The Easyhaler^®^ is another handy inhaler for daily use in individuals with asthma and COPD [68].

## 3. Clinical Trials of Inhaled Drug Delivery

Despite extensive preclinical success with inhaling nanocarriers for the treatment of lung cancer, few have progressed to human testing. One notable example is aerosolized liposomal 9-nitro camptothecin (9NC), which in a Phase I trial achieved 4–10× higher lung drug levels than plasma, exhibited manageable systemic toxicity at doses up to 20 µg/kg/day, and produced preliminary tumor responses—leading to a recommended Phase II dose of 13.3 µg/kg/day [69]. The liposomal formulation has shown promise for the pulmonary delivery of anticancer drugs in the treatment of lung cancer. These nanocarriers can enhance drug targeting, reduce systemic toxicity, and provide sustained release in the lungs [70]. Liposomes can effectively entrap various therapeutic agents and deliver them to the peripheral airways using nebulizers, although sharing during aerosolization may affect stability [71]. Affect stability Clinical trials have demonstrated the potential of inhaled liposomal formulations, such as Arikace^®^ and Pulmaquin^®^, for treating lung infections [71]. The sustained-release lipid inhalation targeting aerosol was produced using a PARI LC^®^ Star nebulizer (PARI Lifescience, Starnberg, Germany), achieving an MMAD of 3.7 µm and a GSD of 1.9 µm. This aerosolized approach showed significantly reduced systemic toxicities (hematotoxicity, neurotoxicity, and nephrotoxicity) compared to oral cisplatin administration, indicating that combining nanocarrier formulations with inhalation delivery effectively lowers adverse systemic effects. Additionally, a Phase I clinical trial evaluated aerosolized liposomal IL-2 in patients with pulmonary metastases [72,73].

The formulation was administered via a Puritan twin-jet nebulizer (Medtronic, Minneapolis, MN, USA) for 20 min, three times daily, over 8 days. Treatments were well-tolerated without significant toxicity, and extensive preclinical studies are needed to reinforce future clinical findings. Aerosolized sustained-release lipid inhalation targeting (SLIT) cisplatin in 17 patients with advanced lung cancer. Liposomal cisplatin was delivered via a PARI LC Star nebulizer (MMAD 3.7 µm), demonstrating localized pulmonary delivery with minimal systemic exposure. Treatment was well-tolerated, with only mild respiratory effects reported. Stable disease was achieved in 12 patients [73], supporting further clinical evaluation as shown in Table 2. Even though nanocarriers have a lot of potential for the management of lung cancer through the pulmonary route [74]. Amikacin Liposome Inhalation Suspension (Arikayce^®^) is the first liposomal nanocarrier approved for human inhalation therapy. Delivered via a vibrating-mesh nebulizer, it achieves high pulmonary drug concentrations with minimal systemic exposure. Its FDA approval in 2018 for treatment-refractory MAC lung disease demonstrates the clinical feasibility of inhalable liposomal formulations, supporting their potential translation to lung cancer therapy, as shown in Table 2 [75]. There are not enough clinical investigations on them yet. As a result, there is a need to broaden their scope of applicability to include clinical trials under suitable ethical guidelines.

## 4. Different Nanomaterials for Pulmonary Drug Delivery

### 4.1. Lipid-Based Nanocarrier for Inhalation

**Figure 2:**
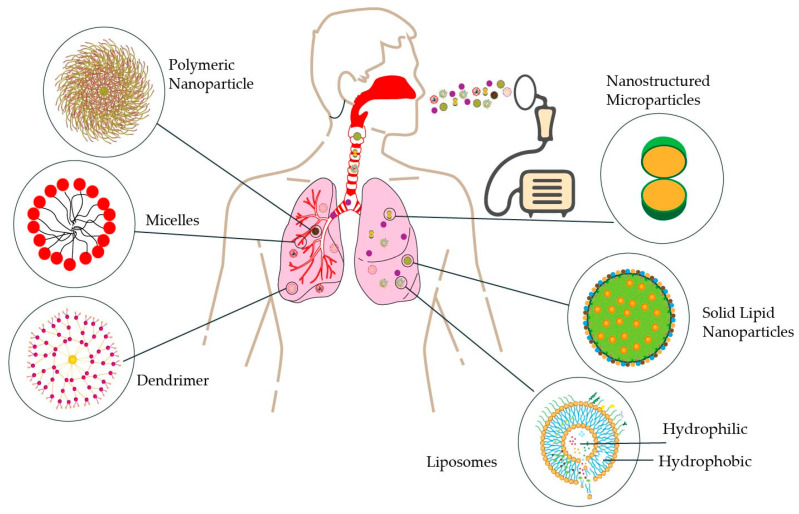
Various types of nanoparticles are used for inhalable lung cancer therapy. This figure illustrates multiple nanoparticle types—polymeric nanoparticles, micelles, dendrimers, liposomes, solid lipid nanoparticles, and microparticles—employed in inhalable lung cancer treatments, showcasing their diverse structures and mechanisms for targeted drug delivery to the lungs.

Lipid-based formulations, particularly those containing phospholipids like endogenous lung surfactants, offer biocompatibility and versatility for pulmonary drug delivery. These formulations can enhance therapeutic efficacy, improve pharmacokinetics, and reduce toxicity [79], As illustrated in Figure 2.

### 4.2. Liposomes

Liposomes have emerged as a leading nano-based drug delivery system, with numerous FDA-approved formulations on the market [80]. These lipid vesicles offer several advantages, including targeted delivery, sustained release, and reduced toxicity [79].

The synthesis method determines the specific type of liposome produced. Liposomes can be categorized into unilamellar vesicles (ULVs) containing a single bilayer membrane, oligolamellar vesicles (OLVs) with 2 to 5 bilayer membranes, and multilamellar vesicles (MLVs) possessing five or more bilayers. ULVs are further subdivided based on size into small unilamellar vesicles (SUVs, 20–100 nm), large unilamellar vesicles (LUVs, 100 nm to 1 µm), and giant unilamellar vesicles (GUVs, >1 µm). Among these, SUVs are most used for drug delivery due to their uniform drug encapsulation, controlled release profiles, and extended circulation times [81].

They have been successfully applied in various therapeutic areas, including cancer, bacterial, and viral diseases [82]. Recent advancements in liposome technology have focused on improving preparation techniques and expanding their application to novel modalities, such as nucleic acid and immunotherapies [80]. The development of targeted liposomal drug delivery systems, particularly for cancer therapy, has been a key area of research [83].

Structurally, liposomes are self-assembled carriers composed of a hydrophobic lipid bilayer enclosing a hydrophilic core, making them ideal for encapsulating water insoluble drugs, particularly chemotherapeutic agents like paclitaxel and doxorubicin [83]. Their size, composition, and stability can be optimized for drug delivery, with recent innovations, such as nano-bowl-supported liposomes, further enhancing stability and delivery efficiency [84]. These properties make liposomes highly promising for cancer treatment, offering active targeting capabilities and reduced off-target toxicity [85]. However, successful clinical translation requires advanced analytical characterization techniques to address the complex attributes of liposomes and lipid nanoparticles [86].

Researchers are developing treatments for liver and lung diseases using synthetic mRNA to replace missing or malfunctioning proteins. In lung cancer treatment, FDA-approved inhalable liposomes, resembling lung components, enhance drug targeting and bioavailability. Ciprofloxacin-loaded liposomes significantly improved these outcomes [87].

PEG-modified unilamellar liposomes loaded with afatinib dimaleate were formulated as a dry powder inhaler (LDPI) for NSCLC. The formulation (181.2 nm, +9.65 mV) demonstrated strong aerodynamic performance (MMAD 2.83 µm, FPF 63.65%, >85% emitted dose), sustained zero-order release across pH 5.5–7.4, and enhanced cytotoxicity in A549 cells (IC_50_: 1.93 µM vs. 2.58 µM for free drug), along with improved cellular uptake, supporting its potential for targeted pulmonary delivery Table 3 [88] Liposomal curcumin dry powder (MMAD 5.81 µm, FPF 46.71%) also exhibited efficient lung deposition, enhanced cytotoxicity and uptake in A549 cells compared to free curcumin and gemcitabine, and modulated several in vivo biomarkers by reducing TNF-α, MDA and increasing caspase-3 expression, thereby supporting its potential as an inhalable lung cancer therapy [89]. Lipid–polymer hybrid nanoparticles (276.9 nm, −10.6 mV) further improved siRNA delivery via inhalation, achieving 84.83% transfection efficiency in A549 cells, outperforming lipoplexes in 3D A549-3T3 spheroids while maintaining stability in bronchoalveolar lavage fluid (BALF) [90].

Erlotinib and genistein were co-loaded into DPPC:Cholesterol:DOPE (72:8:20 mol%) liposomes (~200 nm, neutral charge) for pulmonary delivery targeting NSCLC. The formulation demonstrated synergistic effects in NSCLC cell lines (H3255, H1650, H1781), achieving a fine particle fraction of up to 70% via air-jet nebulization, which offers efficient lung deposition with potential for localized lung cancer therapy while minimizing systemic toxicity [29]. Inhalable liposomal DPI for NSCLC also demonstrated improved lung deposition (MMAD 3.15 µm, FPF 83.71%), sustained release up to 72 h, enhanced bioavailability inferred from improved lung deposition and dissolution, and reduced systemic toxicity [91]. Furthermore, paclitaxel-loaded conventional liposomes (SPC: Cholesterol, 64.3 nm, −9.96 mV) were encapsulated into *E. coli* and *L. casei* (LPB system) for inhaled lung cancer therapy, resulting in high A549 cell uptake, strong apoptosis (98.6%), tumor inhibition in lung cancer rat models, immune activation, and selective lung targeting [8]. Liposomal gefitinib DPI (SPC: Cholesterol, 69.8 nm, −9.67 mV; MMAD 7.83 µm, FPF 33.8%) achieved 2.36-fold higher bioavailability compared to oral delivery, improved lung deposition, tumor reduction, apoptosis induction, and inflammation suppression in primary lung cancer rat models [92]. Rolapitant-loaded nanovesicles (liposomes: 225.1 nm, –24.5 mV) enhanced cytotoxicity, cellular uptake, and permeability in A549 lung cancer cells, inhibited NK-1 receptors and achieved favorable lung biodistribution following intratracheal administration [93]. Finally, spray-dried vincristine-loaded liposomes (112.6 nm, −21.56 mV) achieved efficient lung delivery (FPF 14.99%), sustained release over 96 h, enhanced cytotoxicity in A549 and MCF-7 cells, improved pharmacokinetics (4.29× AUC, 6.3× Cmax, prolonged t½), and reduced metabolism through minimal CYP3A4 activity, offering a promising inhalable lung cancer therapy [94].

### 4.3. Solid Lipid Nanoparticles

Following liposomes, Solid Lipid Nanoparticles (SLNs) have emerged as prominent nanocarriers for inhalation therapy. Their capacity for efficiently entrapping hydrophobic therapeutic agents, coupled with robust scalability for industrial production, has driven an increased research focus on SLNs in recent years, particularly for cancer applications. Notably, SLN manufacturing utilizes an emulsion system comprising a blend of solid lipids, allowing for synthesis under ambient temperature conditions [95]. Folate-targeted SLNs enabled efficient paclitaxel (PTX) delivery to “folate receptor” FR-overexpressing lung tumors with 32× higher lung retention and sustained release. They demonstrated undetectable systemic exposure and 6-fold greater anti-cancer efficacy in M109-HiFR cells compared to inhaled Taxol. They depict a promising inhalable nanocarrier strategy for targeted, sustained, and safer lung cancer therapy. Refer to Table 3 [96].

Despite their benefits, SLNs have drawbacks, such as limited drug loading and crystallization during storage, which have been addressed by the development of nanostructured lipid carriers (NLCs) [97]. The discovery of nanostructured lipid carriers (NLCs) overcame some of these problems. Offering improved drug release modulation [97]. The self-assembly of SLNs from spray-dried microparticles has been demonstrated, providing a non-toxic delivery platform for various drugs [98]. Overall, SLNs and NLCs are considered safe and effective drug delivery systems with potential for further development [99]. Rapamycin-loaded SLNs (mean size: 237.5 nm; MMAD: 5.4 μm) were successfully developed using 5% *w*/*w* mannitol. These SLNs inhibited mTOR signaling comparably to free rapamycin and significantly reduced proliferation in TSC2-deficient mouse embryonic fibroblasts, confirming bioactivity. Concurrently, folate-grafted chitosan-PEG copolymer surface-engineered solid lipid nanoparticles (SLNs) were designed for inhalable paclitaxel delivery to lung tumors. Both systems demonstrate the potential of SLNs as biocompatible, industrially scalable nanocarriers for pulmonary therapy Refer Table 3 [96]. These were prepared using high-shear homogenization and characterized via DSC, FTIR, and particle size analysis. Optimized SLNs exhibited spherical morphology with suitable aerodynamic properties and demonstrated potent anticancer activity against A549 cells. Please refer to Table 3 [100].

Thin-film freeze-drying (TFFD) was used to assess the possibility of creating aerosolized dry powders of lipid nanoparticles for pulmonary medicine delivery, including small interfering RNA (siRNA). The study concluded that TFFD represents a promising method for this purpose, with excellent aerosol performance (fine particle fraction: 37 %, MMAD: 3.96 µm) for deep-lung delivery, and demonstrated mucus-layer penetration, as shown in Table 3 [101]. Fav-SLNps (Favipiravir-loaded solid lipid nanoparticles) were successfully developed and shown to be safe for A549 cells. They exhibited anti-proliferative effects, induced necrosis, and enhanced macrophage uptake compared to free drugs, suggesting potential lung cancer treatment [102]. Commending the potential of solid lipid nanoparticles (SLN) and nanostructured lipid carriers (NLC) in pulmonary drug delivery, their ability to offer prolonged release, low toxicity, and improved stability during nebulization [102].

**Table 3:** Lipid-Based Nanocarrier for Inhalation.

Engineered Nanoparticles	Drugs	Physical Parameters	Performance	Ref.
Liposomes	Paclitaxel	Size: 64.3 ± 2.4 nm ZP: −9 nm ± 0.48 mV MMAD: N/A FPF: N/A	Inhalable paclitaxel-in-liposome-in-bacteria system for primary lung cancer; demonstrated high lung targeting, enhanced cellular uptake (A549 cells), strong apoptosis induction (up to 98.6%), significant tumor suppression in lung cancer rat model, immune activation (↑ TNF-α, IL-4, IFN-γ), and selective lung biodistribution after pulmonary delivery…	[8]
PEGylated Liposomes	Afatinib Di maleate	Size: 181.2 ± 5.0 nm ZP: +9.65 ± 0.32 mV MMAD: 2.83 µm FPF: 63.65%	Polyethylene glycol (PEG)-modified unilamellar liposomal dry powder showed high lung deposition (>85% emitted dose), MMAD 2.83 µm, FPF 63.65%, sustained release across pH 5.5–7.4 (zero-order kinetics), enhanced cytotoxicity, and improved cellular uptake in A549 cells	[88]
Liposomes	Curcumin	Size: 94.65 ± 22.01 nm ZP: N/A MMAD: 5.81 µm FPF: 46.71%	Liposomal dry powder with an MMAD of 5.81 µm and an FPF of 46.71% showed efficient lung deposition. In A549 cells, it outperformed free curcumin and gemcitabine in cytotoxicity, indicating strong potential for lung cancer treatment.	[89]
PEGylated Liposomes	siRNA	Size: 276.9 ± 4.8 nm ZP: −10.6 ± 0.8 mV MMAD: N/A FPF: N/A	PLGA-based hybrid nanoparticles enhanced mucus penetration, Bronchoalveolar Lavage Fluid BALF stability, and A549 cell uptake. Showed 84.83% transfection and superio r gene silencing in 3D spheroids and improved cellular uptake A549-3T3	[90]
Liposomes	Erlotinib + Genistein	Size: 200 nm ZP: ~0 mV Neutral MMAD: N/A FPF: N/A	Pulmonary delivery for NSCLC; tested on H3255, H1650, and H1781 cell lines; local inhalation enhances lung deposition and reduces systemic toxicity	[91]
Liposomes	Gefitinib	Size: 69.82 ± 2.60 nm ZP: −9.67 ± 0.73 mV MMAD: 7.837 µm; FPF: 33.81	Inhalable liposomamV MMADr primary lung cancer demonstrated high lung distribution, rapid absorption, 2.36-fold higher bioavailability vs. oral, significant tumor reduction, apoptosis induction (Caspase-3, TUNEL), P-Akt suppression, inflammation attenuation (↓ TNF-α, W/D ratio); tested in primary lung cancer rat model.	[92]
Liposomes	Rolapitant (RP) FDA- approved drug	Size: 25.1 ± 7.3 nm ZP: −24.5 ± 1.4 mV MMAD: N/A FPF: N/A	Inhalable nanocarrier formulations for lung cancer tested on A549 lung cancer cells showed enhanced cytotoxicity, improved permeability, high cellular uptake, NK-1 receptor inhibition, and favorable biodistribution to lungs after intratracheal administration.	[93]
Liposomes	Vincristine (VCR)	Size: 112.6 ± 3.7 nm ZP: −21.56 ± 2.53 mV MMAD: N/A FPF: 14.99 ± 0.27%	Inhalable liposomal spray-dried powder for lung cancer; sustained release (65% over 96 h), enhanced cytotoxicity against MCF-7 and A549 cells (IC50: 24.4 nM & 55.3 nM), improved lung targeting, lower hepatic metabolism (due to CYP3A4 deficiency), higher AUC and Cmax, prolonged t1/2, and reduced clearance in rat model.	[94]
SLNs	Paclitaxel (PTX)	Size: 249 ± 36 nm ZP: +32 ± 1 mV MMAD: N/A FPF: N/A	Targeted delivery to folate receptor (FR)-overexpressing lung tumors via folate-mediated endocytosis using solid lipid nanoparticles (SLNs) loaded with paclitaxel (PTX) achieved 32× higher lung retention, undetectable systemic exposure, sustained PTX release (50% over 72 h), and enhanced anti-proliferative efficacy in M109-HiFR cells.	[96]
SLNs	Curcumin	Size:14.7 nm ZP: −22.5mV MMAD: N/A FPF: N/A	Enhanced curcumin solubility and dissolution rate in lung tissue; high encapsulation (90.2%) and drug loading (8.56%); maintained aerodynamic diameter (~4.03 μm) for deep lung deposition; sustained-release in simulated lung fluid; reduced cytotoxicity in BEAS-2B cells—indicating a safe, effective dry-powder inhalation system	[100]
SLNs	TNF-α siRNA	Size:164.5 ± 28.3 nm ZP: −29.1 ± 8.6 mV Mv MMAD: 3.96 µm FPF: 37%	Thin-film freeze-drying yields a porous, brittle dry powder that preserves SLN size and charge, retains siRNA function (effective TNF-α knockdown in macrophages), and exhibits excellent aerosol performance (fine particle fraction 37%, MMAD 3.96 µm) for deep-lung delivery, plus demonstrated mucus-layer penetration.	[101]
SLNs	Favipiravir	Size:: 693.1 ± 40.3 nm ZP: −13.3 ± 0.3 MMAD:3.0 ± 0.4 µm FPF: 60.2 ± 1.7%	Favipiravir-loaded SLNs (~693 nm; −13.3 mV) prepared via hot-evaporation nebulized into a respirable aerosol (FPF 60.2%, MMAD 3.0 µm, GSD 2.33). They were biocompatible up to 322.6 µg/mL in A549 cells, inducing cell-cycle arrest, necrosis, and autophagy inhibition, as well as a 1.23-fold increase in macrophage uptake, for effective pulmonary anticancer delivery.	[103]
Liposomes	sorafenib tosylate (ST)	Size: 111.15 ± 1.03 nm ZP: −29.87 ± 0.56 mV MAD: 3.15 ± 0.42 µm FPF: 83.71 ± 2.09%	Iµm.lable liposomal DPI for NSCLC demonstrated improved lung deposition (MMAD 3.15 µm, FPF 83.71%), sustained release up to 72 h, potentially enhanced bioavailability inferred from improved lung deposition and dissolution, and reduced systemic toxicity.	[104]

### 4.4. Polymeric Nanocarriers for Inhalation

Over the past decade, polymer-based nanotherapeutics have garnered significant attention due to their unique structural versatility and efficacy in delivering therapeutic agents, particularly for cancer chemotherapy. These polymeric nanoparticles (Figure 2) enable high drug loading capacity, protection against degradation, and controlled site-specific release, collectively enhancing bioavailability while minimizing systemic adverse effects [105].

### 4.5. Polymeric Nanoparticles

Poly(lactide-co-glycolide) (PLGA), a noncytotoxic and biocompatible polymer, is frequently utilized in drug delivery systems due to its capacity for prolonged release. Anti-cancer medications have been delivered with success using recent advancements in the use of Polyethylene glycol (PEG)-PLGA nanoparticles for cancer treatment [106,107]. Elbatanony et al. employed the nanoprecipitation method to prepare inhalable PLGA nanoparticles encapsulating afatinib—a first-line NSCLC therapy—and assessed their in vitro activity against KRAS-mutant A549 and H460 cell lines [108]. Over the past few decades, researchers have engineered and functionalized these nanoparticles to enable site-specific targeted drug delivery in NSCLC [109].

Vanza et al. formulated afatinib-loaded inhalable PLGA nanoparticles via a double-emulsion solvent-evaporation method optimized by a 3^2^ factorial design, achieving an entrapment efficiency of 78.15 ± 2.20% and a particle size of 198.1 ± 3.5 nm. Scale-up to a 75 mL batch preserved these critical quality attributes. Lyophilization with trehalose and L-leucine produced an inhalable dry powder (6.883 ± 0.369 µm) with 77.86 ± 3.02 % drug content, >85% deep-lung deposition, and sustained release (>80% over 18–34 h across pH 5.5–7.4). In A549 cells, the formulation exhibited enhanced cytotoxicity (IC_50_ 1.573 ± 0.067 µM vs. 2.576 ± 0.110 µM for free drug) and improved cellular uptake while remaining stable under intermediate and long-term storage conditions, demonstrating strong potential for targeted pulmonary delivery in NSCLC therapy [110].

Using a QbD-guided approach, Bardoliwala et al. optimized PLHNCs co-loaded with Docetaxel and ABCB1 shRNA to achieve high drug/gene loading, a favorable size and charge for enhanced uptake, and excellent aerosol performance (deep-lung deposition), positioning this system as a promising inhalable therapy to overcome MDR in lung cancer [111]. These studies optimized the parameters for spray-dried powders containing nucleic acid-PEI nanoparticles, preserving bioactivity and enabling reconstitution of the nanoparticles. The versatility and efficiency of PLGA nanoparticles have been demonstrated in various disease models, including the successful delivery of tacrine from the nose to the brain and lung targeting of silibinin and moxifloxacin. Including effective tacrine delivery from the nose to the brain and lung targeting silibinin and moxifloxacin [112,113,114]. Researchers have successfully functionalized PLGA nanoparticles with an anti-HER2 affibody for selective binding to cancer cells that overexpress the HER2 protein. Furthermore, the potential of TRAIL therapy in cancer treatment has been explored, with strategies to enhance its anticancer activities and identify substances that sensitize tumor cells to TRAIL-induced apoptosis [114,115].

### 4.6. Micelles

Micelles, colloidal particles made of amphiphilic block copolymers or surface-active chemicals, have been widely researched for their potential in drug administration [116]. Highlights their unique qualities, such as increased bioavailability and the potential to be changed for tailored Medication administration [107]. Further explores the structural details and stabilities of these micelles, with a focus on the impact of cyclic topology on their formation and integrity [117]. Amphiphilic polysaccharide copolymer with micellar nanoparticles self-assembles in water, stabilizes curcumin, and exhibits low toxicity [117]. However, their stability in biological fluids and interaction with the biological environment are key challenges [118]. To enhance the drug-encapsulating capacity and stability, it is essential to consider the compatibility between the drug and the polymer. And drug-release kinetics [119]. Despite these challenges, Polymeric micelles have shown improvements in Pharmacokinetic characteristics and improved efficacy in preclinical and clinical studies [120]. Ciprofloxacin-coated nano micelles prolong pulmonary residence and bolster antibacterial efficacy; when spray-dried into nano aggregates optimized by factorial design, they retain stability and enhance drug performance in dry-powder inhalers [121]. Omalizumab is encapsulated in polymerases for targeted delivery to the lungs, with PEG-polyester nanoplatforms ensuring optimal absorption and cell uptake through spherical micelles [122]. Polymeric micelles, ~17 nm in size with near-neutral surface charge (ζ = 0.37 mV), achieved high co-loading of doxorubicin and curcumin, effectively overcame multidrug resistance in lung cancer cells through enhanced uptake and synergistic cytotoxicity, sustained drug release, prolonged blood circulation, and robust tumor growth inhibition in A549 cell lines Table 4 [123]. The effects of a magneto-micelle suspension on rat lungs were investigated over 40 days. Hemodynamic changes in lung tissue were observed initially but diminished over time. Accumulation of magneto micelles in the lungs did not cause harm to pneumocytes, macrophages, or the blood-air barrier [124].

### 4.7. Nanostructured Microparticles

Microparticles, designed explicitly for pulmonary drug delivery, present a viable means of resolving the issues related to traditional inhalation treatments. These particles, typically ranging from 1–3 μm in size and density of around 1 g/cm^3^, aggregate in dry powder inhalers, clearing quickly by lung macrophages. To address these challenges, studies have explored the use of carrier-based formulations and the development of nano-structured microparticles for inhalation [125]. These particles, which are a combination of nanoscale drug carriers, have been found to consistently and persistently deliver medication to the airways after inhalation [83,126]. Furthermore, the use of non-mucoadhesive, such as mucus-penetrating particles, has been suggested to enhance pulmonary drug delivery [127]. The emulsification technique produces porous PLGA particles for efficient lung delivery, which resist phagocytosis [128]. PLGA is a widely studied polymer for the preparation of sustained-release microspheres for pulmonary administration [129]. Docetaxel-loaded PLGA-PLX-188 nanoparticles (~222 nm; −34.8 mV) were successfully spray-dried into nano-embedded microparticles that aerosolized into a DPI with an MMAD of 3.74 µm and FPF of 42.96 %. Upon deposition, the microparticles released ~47.8 % of redispersed NPs retaining their size, and PDI provided sustained drug release over 144 h, remained stable for at least three months, and delivered significantly greater cytotoxicity in A549 cells due to improved nanoparticle uptake—demonstrating strong potential for targeted inhalation therapy in NSCLC [130].

**Table 4:** Polymeric nanocarriers for delivery.

Engineered Nanoparticles	Drugs	Physical Parameters	Performance	Ref.
PLGA NPs	Afatinib	Size: 180.2 ± 15.6 nm ZP: −23.1 ± 0.2 mV MMAD: 4.7 ± 0.1 µm FPF: 77.8 ± 4.3%	Afatinib-loaded PLGA nanoparticles achieved 34.4 ± 2.3% entrapment with sustained release (56.8 ± 6.4% over 48 h) and excellent inhalable properties (MMAD 4.7 ± 0.1 µm; FPF 77.8 ± 4.3%). They outperformed free afatinib in KRAS-mutant A549 and H460 cells by enhancing cytotoxicity, cellular uptake, and penetration–growth inhibition in 3D tumor spheroids.	[108]
PLGA NPs	Afatinib-loaded PLGA nanoparticles dry powder inhaler	Size: 198.1 ± 3.5 nm ZP: −0.519 ± 0.197 mV MMAD: N/A FPF: N/A	The inhalable dry powder of afatinib-loaded PLGA nanoparticles achieved high entrapment (78.2 ± 2.2%) and drug loading (3.90 ± 0.11%), formed a respirable powder (~6.9 µm) with good flow and >85 % deep-lung deposition, provided sustained release (>80% over 18–34 h across pH 5.5–7.4), and markedly improved cytotoxicity and cellular uptake in A549 cells versus the free drug.	[110]
Polymeric–Lipid Hybrid Nanocarriers	Docetaxel + ABCB1 shRNA pDNA	Size: 124.1 ± 1.9 nm ZP: +26.5 ± 1.8 mV Mv MMAD: N/A FPF: N/A	High Docetaxel encapsulation (85.6 ± 2.8%) and shRNA complexation (97.4 ± 1.6%). Nanoscale size (<200 nm) and positive surface charge enhance cellular uptake and avoid rapid clearance. Processed into a respirable dry powder (MMAD 3.56 ± 0.21 μm; FPF 68.3 ± 2.5%) for deep-lung delivery, demonstrating potential to reverse multidrug resistance in NSCLC	[111]
Polymeric micelles	Doxorubicin + Curcumin	Size: 17.02 ± 2.58 nm ZP: 0.37 ± 0.014 mV MMAD: N/A FPF: N/A	Synergistically reversed doxorubicin resistance in A549/cells (IC_50_ 3.95 µg/mL) via energy-dependent, caveolae-mediated uptake; Provided sustained release and prolonged systemic circulation with elevated plasma levels over six h. Significantly inhibited tumor growth in Lewis lung carcinoma–bearing mice	[123]
Microparticles	DocetaxelVenza	Size: 222.1 nm ZP: −34.8 mV MMAD: 3.74 µm FPF: 42.96%	Docetaxel-loaded PLGA-PLX-188 nanoparticles (~222 nm; −34.8 mV) were successfully spray-dried into nano-embedded microparticles that aerosolized into a DPI with an MMAD of 3.74 µm and FPF of 42.96%. Upon deposition, the microparticles released ~47.8% of the redispersed NPs, retaining their size and polydispersity index (PDI), which provided sustained drug release over 144 h. The microparticles remained stable for at least three months and delivered significantly greater cytotoxicity in A549 cells due to improved nanoparticle uptake, demonstrating strong potential for targeted inhalation therapy in non-small cell lung cancer (NSCLC).	[130]

### 4.8. Dendrimers

A type of hyperbranched homopolymer has a unique structure with an initiator core, interior layers, terminal surface groups, and drug-entrapping spaces, resembling unimolecular micelles [131]. This structure allows them to be effective drug carriers, with the ability to carry both hydrophobic and hydrophilic drugs and to be modified for specific drug delivery purposes [132]. Their nanoscopic size, narrow polydispersity index, and multiple functional groups make them versatile and attractive for drug delivery applications [133]. TPP-modified dendrimers were examined to improve SIRNA transfection in the lungs. Epithelium. Elevated TPP density correlated with enhanced gene knockdown. Mannitol microparticles containing dendriplexes are efficiently delivered to deep lung regions [134]. Pulmonary drug delivery surpasses systemic administration for lung diseases. Poly(amidoamine) (PAMAM) dendrimer interaction with pulmonary surfactant using Langmuir monolayers and molecular dynamics simulations. Results suggest that PAMAM dendrimers may be potential respiratory drug nanocarriers [135]. Dendrimers, a type of polymeric nanoarchitecture, have shown great potential in drug delivery and targeting due to their unique properties. They can enhance drug solubility, stability, and bioavailability, as well as shield tissues from toxicity [136]. The use of dendrimers as respiratory nanocarriers has also been investigated, yielding promising results in terms of their capacity to permeate the lipid monolayer and their potential as drug nanocarriers [135].

### 4.9. Lipid vs. Polymeric Nanocarriers: Tailoring Pulmonary Drug Delivery Systems

Lipid-based nanocarriers have emerged as promising vehicles for pulmonary drug delivery, particularly for glucocorticoids and anticancer drugs [137]. These nanocarriers offer advantages such as high biocompatibility, improved drug loading, and enhanced lung retention compared to non-lipid-based alternatives [138]. Surface modification of lipid nanocarriers can further enhance their targeting efficiency and controlled release properties [138]. However, successful inhalation therapy depends on various factors, including the physicochemical properties of the payload, formulation, and interaction with lung fluids [139]. While both lipid and polymer-based nanocarriers show potential for pulmonary drug delivery, lipid-based systems demonstrate superior lung accumulation and retention after inhalation [140].

## 5. Challenges

In recent decades, nanoparticle-based medicines have advanced rapidly, showing promise in cancer diagnosis, imaging, and treatment, including for the treatment of lung cancer. These nanoparticles can be customized for personalized therapy, often combining different components to encapsulate drugs and target specific sites. However, translating these innovations into clinical inhalation therapies for lung cancer faces challenges in synthesizing nanoparticles with ideal properties and establishing standardized testing protocols due to their complex nature. Nanoparticle-based medicines have shown great potential in the diagnosis, imaging, and treatment of lung cancer; however, their translation into clinical inhalation therapies faces significant challenges [141].

### 5.1. Clearance of Nanoparticles

**Figure 3:**
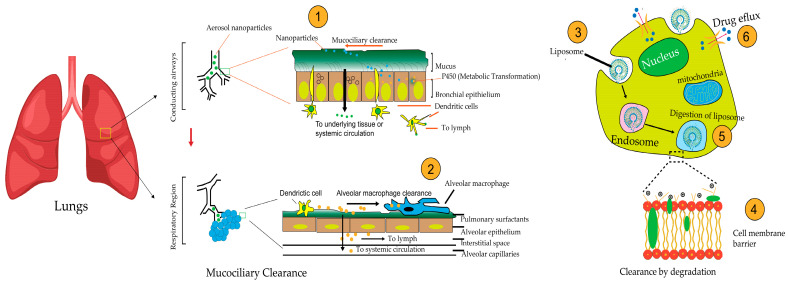
Mechanisms of Nanoparticle Clearance. The figure illustrates how inhaled nanoparticles are cleared (1) Mucociliary clearance in the conducting airways removes inhaled particles via coordinated mucus and cilia movement. (2) Phagocytosis by alveolar macrophages engulfs and eliminates nanoparticles in the respiratory region, (3) Enzymatic degradation occurs within endo-lysosomal compartments, and (4) Endosomal escape allows some nanoparticles to bypass degradation. (5) Lysosomal degradation further digests nanoparticles within lysosomes. (6) Drug efflux through transporter proteins pumps nanoparticles or their released drugs out of cells. These barriers collectively impact nanoparticle deposition, retention, and therapeutic efficacy in lung cancer treatment.

Nanoparticle clearance in the respiratory region is influenced by particle size, shape, and delivery method. In the tracheobronchial system, which serves as conductive airways and the respiratory region, as illustrated in Figure 3, the clearance of nanoparticles varies based on geometry, with 24-h retention ranging from 72.7% to 87.0% for different shapes [142]. Particle size significantly affects deposition, with a 67.5% decrease in deposition fraction as size increases from 10 nm to 100 nm [143]. Exposure path selection impacts nanoparticle deposition, with distinct values observed for different respiratory routes [143]. Gold ultrasmall nanoparticles administered intranasally can reach the lung parenchyma and secondary organs, with almost complete excretion within 10 days [144]. Pulmonary delivery of nanoparticle chemotherapy for lung cancer treatment holds promise but faces challenges due to the physiology of the respiratory tract and lung clearance mechanisms [145].

### 5.2. Mucociliary Clearance

Mucociliary clearance (MCC) is a key defense mechanism in the respiratory system, utilizing coordinated mucus and cilia action to remove inhaled particles and pathogens. Ciliated cells, equipped with 100–250 motile cilia beating at 12–15 Hz, propel the mucus layer at 8–10 mm/h toward the pharynx, as illustrated in Figure 3 [23,146]. Additionally, NPs can disrupt cellular metabolism in bronchial epithelial cells, with nano polystyrene inducing autophagy and endoplasmic reticulum stress, while nano ZnO causes ROS-mediated cell death and mitochondrial dysfunction [147]. Nanoparticles have difficulty adhering to airways due to the presence of mucus, prompting the development of methods to enhance penetration, which requires thorough evaluation for clinical safety and relevance [148]. Rapid mucociliary clearance significantly decreases the effective lung dose, with fast TiO2-nanoparticle clearance accounting for 30% of the instilled dose within 1 h [149]. The effectiveness of mucoadhesive particles (MAP) for delivering drugs to the lungs is being questioned, and there is a suggestion that nonstick mucus-penetrating particles (MPP) might be more beneficial in terms of retention and distribution within the lungs. This idea is supported by evidence [127]. This discovery holds immense importance for delivering drugs or genetic material within the cells of the underlying epithelial tissue. It highlights the crucial role of physicochemical attributes, such as particle size and surface properties, in inhaled nanoparticles [150]. Particle penetration through mucus relies on size; mucin forms a network filtering size. Larger particles are impeded, while smaller ones could theoretically diffuse through, considering the mesh pore size [151]. Inspired by mucus-penetrating viruses, researchers have discovered zwitterionic nanoparticles with a net neutral charge, which reduce the adsorption of biomolecules to their surface [152,153]. Although rare in pulmonary drug delivery studies, zwitterion-functionalized nanoparticles are widely used in oral drug delivery, suggesting potential future advancements [153].

### 5.3. Influence of Surface Hydrophobicity

The surface properties of nanoparticles significantly influence their ability to penetrate mucus and navigate the respiratory system. Studies have shown that hydrophilicity, surface charge, and particle size are crucial factors affecting mucus penetration [154]. Hydrophilic particles penetrate mucus more effectively than hydrophobic ones, indicating that surface engineering to reduce particle-mucus adhesion could be beneficial [155]. One approach to improving mucus penetration involves coating nanoparticles with polyethylene glycol (PEG). Higher molecular weight (MW) PEG chains can alter mucin interactions, facilitating better mucus penetration [156]. The use of low molecular weight (2–5 kDa) PEG to facilitate airway mucus penetration has been supported by several studies [157]. PEGylation, the process of attaching polyethylene glycol (PEG) to liposomes, has been a key strategy in research to evade mucous clearance during inhalation [158]. Hydrophilic PEG-coated PLGA nanoparticles exhibited prolonged retention in lung mucus after inhalation compared to non-coated PLGA nanoparticles [127]. Conversely, increasing hydrophobicity in PEGylated dendrimers has been shown to double mucociliary clearance in rats compared to fully PEGylated ones [159]. While PEGylation is widely used for particle surface modification, it can lead to issues such as the formation of anti-PEG antibodies, which can trap particles in mucus and affect cellular uptake [151]. Alternative surface modifications, such as poly(2-alkyl-2-oxazolines) and mucolytic enzymes, may also be effective in enhancing mucus permeation of nanoparticles [152]. As illustrates Figure 3, PEGylation plays a role in improving mucus penetration, while the challenges of hydrophobic nanoparticles impact their deposition and absorption.

### 5.4. Alveolar Macrophage Clearance

Alveolar macrophages play a crucial role in clearing inhaled particles from the lung, with their phagocytic behavior being influenced by particle size, charge, and modification [160]. As illustrated in Figure 3, these macrophages interact with inhaled nanoparticles, with their phagocytic activity significantly influenced by the particle’s physical and chemical properties.

Particle size affects pulmonary delivery. Only particles between 1–5 mm aerodynamic diameter are inhalable, which is crucial for therapeutic agent delivery [161]. Figure 3, highlights how particle size plays a pivotal role in determining the efficiency of pulmonary delivery and macrophage uptake. Tumor vaccines had been delivered to lung dendritic cells via cationic nanoliposomes. Although liposomes effectively entrapped antigens, their release was limited. DOTAP liposomes were deemed safe and predominantly internalized by alveolar macrophages in mice, impeding dendritic cell access [162]. The findings suggest that nanoparticles effectively evade alveolar macrophage uptake. Yet, ensuring sufficient lung deposition poses a significant challenge due to rapid exhalation [163]. Recent research produced resveratrol as a nanosuspension spray-dried with mannitol to form nanosuspension-in-microparticles (NS-in-MPs). Compared to the resveratrol-lactose mixture, NS-in-MPs exhibited superior aerodynamic performance and lung [164]. Gold nanoparticles (Au-NPs) with varying surface charges were administered intranasally to mice. Positively charged NH2-PVA Au-NPs were preferentially phagocytosed by alveolar macrophages compared to negatively charged COOH-PVA Au-NPs [165]. Alveolar macrophages are crucial in lung immunity. Pro-inflammatory M1 macrophages are targeted with nanoparticles conjugated with hyaluronic acid and poly (ethylene glycol). Enhanced targeting efficiency toward pro-inflammatory macrophages while improving mucus mobility for better delivery to target sites in the lungs [166].

### 5.5. Clearance by Degradation

Degradation contributes to nanoparticle clearance from the lungs [167]. Despite having a lower metabolic capacity than the liver, the lung contains enzymes that contribute to drug elimination [168]. Figure 3 explains how enzyme P450 metabolizes nanoparticles. Xenobiotic metabolism is crucial for inhaled compounds, involving enzymes such as cytochromes P450 and Phase II enzymes [169]. Inhaled drugs undergo significant pulmonary metabolism, which can impact their safety and effectiveness. Studies compare lung microsomes and cells for in vitro research. However, metabolic clearance in the lung is generally low, with human alveolar type II cells showing lower rates than liver cells [170]. Therefore, mucus production by epithelial cells serves as a physical barrier and a rate-limiting step for the targeting of inhalable drugs [171]. Inhaled medicines may be trapped within the mucus and subsequently removed with multiple clearance mechanisms. Adhesion interactions usually occur between mucus and drug particles via electrostatic, hydrophobic, and hydrogen bonding [172].

The significant roles of mucociliary agents are (i) to act as mechanical filters to entrap particles in the surface liquid onto the airway epithelium and clear by ciliary action. Most insoluble particles with aerodynamic diameters larger than six μm are eliminated by mucociliary clearance. Meanwhile, nano-sized particles can travel faster to reach the bronchial epithelial region and escape the action, (ii) to provide antioxidant activities with its surface liquid, and (iii) to provide a surface biological interaction between microorganisms with luminal inflammation cells to prevent bacterial migration to airway epithelial cells. The clearance mechanism is facilitated by hair-shaped structures like cilia, which are present on the top side of epithelial cells. Once foreign particles are trapped in mucus, cilia beat in a coordinated direction (in the pharynx) to remove the debris either by coughing or swallowing (extensively reviewed in [150]. The study developed inhalable mRNA lipid nanoparticles (LNPs) using leucine and mannitol, achieving effective lung delivery with low toxicity [173].

### 5.6. Challenges and Obstacles to Clinical Implementation

#### Scaling up Inhalable Nanoparticle Production: Challenges Ahead

The scalable, controlled, and reproducible manufacturing of nanomedicines under good manufacturing practice (GMP) conditions presents unique challenges. These challenges include the need for reproducible manufacturing and scale-up, as well as the development of appropriate characterization methods, and the potential impact of minor variations on safety and efficacy. The complexity of nanoparticles requires careful design and engineering, as well as detailed analysis methods. Regulatory considerations, such as those from the FDA and EMA, are also crucial, with a focus on product quality and safety assessment. The development of a manufacturing technique that allows for the transfer of nanomedicine production from the laboratory to an industrial scale is a key barrier, particularly in the case of PLGA-based nanomedicines, highlighted by [174,175]. The development of PLGA-based nanomedicines for industrial-scale up faces several challenges, including batch-to-batch variations and a lack of product consistency. Inline sonication and tangential flow filtration (TFF) have been proposed as scalable and robust manufacturing techniques for PLGA nanoparticles, with the potential for fully continuous operation [176]. These properties include size, shape, composition, crystallinity, drug loading, drug release, and surface functionality and chemistry [177]. This process can be facilitated by techniques such as Flash Nanoprecipitation (FNP) and spray drying, which have been shown to maintain consistent properties across different scales [178]. Key considerations for scaling up the manufacturing of nanotherapeutics include sourcing raw materials, selecting synthesis routes, conducting stability checks, and employing suitable analytical methods [179] The green synthesis of nanoparticles from plant-derived materials can enhance reproducibility and scale-up, but it also presents challenges, such as toxicity [180]. It reviews the techniques for formulating nanoemulsion drug delivery systems, emphasizing the energy requirements and nature of phase inversion [181]. The scale-up of nanomedicines from lab to industrial manufacturing presents significant challenges, as highlighted by [182]. These challenges include maintaining control over the properties of the nanoparticles and ensuring consistency from batch to batch. The use of 3D printing in the development of nanomedicines, as discussed by [183], offers a potential solution to these challenges by enabling the fabrication of tailor-made nanomedicines [184]. further explores the potential of microbial biosynthesis for the green biocatalytic synthesis of nanoscale materials, which could also address the challenges of scale-up in nanomedicine manufacturing.

### 5.7. Obstacles in Screening, Quality Management, and Characterization

Nanomedicine’s physicochemical traits (e.g., size, solubility, stability) are crucial for product performance. Early characterization establishes formulation parameters and their impact on product properties [185]. Identifying methods to characterize nanocarrier properties is technically challenging and poses regulatory compliance issues. Preclinical assessment includes detailed physicochemical descriptions, manufacturing, quality, efficacy, safety, and stability [186]. The lack of reliable and validated techniques for analyzing the characteristics and stability of nanomedicines under Good Manufacturing Practice (GMP) conditions is a significant obstacle to their clinical translation [187]. The limited use of advanced analytical techniques for nanomedicine characterization in industries is a considerable challenge, as these techniques are crucial for understanding the behavior of nano-systems in physiological environments [188]. For a comprehensive analysis, nanomedicine requires up-to-date techniques such as dynamic light scattering (DLS), nanoparticle tracking analysis (NTA), Zetasizer, transmission electron microscopy (TEM)/scanning electron microscopy (SEM), small-angle X-ray diffraction, X-ray photoelectron spectroscopy/FTIR spectroscopy, nuclear magnetic resonance (NMR) spectroscopy, and liquid chromatography (LC) [187]. These techniques are indispensable for investigating how nanomedicines interact with biological systems, their cytotoxic effects, and their properties related to drug delivery [189,190]. This significantly increases nanomanufacturing costs. Specific techniques have constraints; for example, DLS is limited by size and shape, and electron microscopy is labor-intensive [191]. These factors impact circulation time, biodistribution, cell uptake, and interactions with cells or tissues, necessitating their evaluation using suitable methods. Nanomedicine characterization often occurs in settings that inadequately replicate the intricate biophysical conditions of human organs and tissues. Due to the complexity of the human body, accurately predicting in vitro–in vivo correlations for nanomedicines is challenging, which impedes their clinical translation [192]. In brief, advancing robust nanomedicine characterization methods is pivotal for product development. The Nanotechnology Characterization Laboratory (NCL), in collaboration with the NCI, NIST, and the FDA, supports preclinical characterization to expedite translation. Its focus is on providing comprehensive characterization to discern critical parameters governing nanomedicine efficacy and safety. Similar initiatives like the European Nanomedicine Characterization Laboratory (EU-NCL) aim to furnish a comprehensive preclinical assay platform encompassing physicochemical and–vivo biological assessments, enabling thorough evaluation of nanomedicine [193]. Standardized protocols from EU-NCL and its US counterpart, the Nanotechnology Characterization Laboratory (NCL), allow robust nanoparticle assessment properties, interactions, and potential hazards [194]. The standardized approach to nanomedicine evaluation ensures compliance with regulatory requirements and facilitates the translation of nanotherapeutics from preclinical development to clinical applications [194].

### 5.8. Challenges in Multistage Delivery and Deep Tumor Penetration of Nanocarriers

The therapeutic success of inhaled nanocarriers in lung cancer is critically limited by a series of biological and physicochemical barriers encountered during the multistage delivery process. Initially, upon inhalation, nanoparticles must traverse the respiratory mucus layer, a viscoelastic barrier composed of mucins and cellular debris that traps and eliminates foreign particulates through hydrophobic and electrostatic interactions. Additionally, mucociliary clearance and uptake by alveolar macrophages rapidly remove particles before they can reach tumor tissues, significantly reducing bioavailability Challenges in Multistage Delivery and Deep Tumor Penetration of Nanocarriers [195]. Advanced strategies, such as inhalable nanoparticle-in-microsphere systems with size-tunable, charge-reversible, and mucus-penetrating properties, have been developed to enhance lung deposition and local bioavailability. These systems act as “Trojan horses,” improving particle mobility in mucus and evading immune recognition [195]. However, even after overcoming airway barriers, nanocarriers must navigate the dense and heterogeneous extracellular matrix (ECM) within lung tumors. The ECM, rich in collagen, fibronectin, and proteoglycans, acts as a structural scaffold but also presents a significant physical barrier that restricts nanoparticle diffusion and uniform drug distribution. Compounded by elevated interstitial fluid pressure and abnormal vasculature, this microenvironment hampers deep penetration of therapeutic agents into the tumor core [196]. Therefore, the rational design of nanocarriers must integrate multistage features—such as mucus penetration, immune evasion, ECM responsiveness, and tumor-targeted drug release—to enhance delivery efficiency. Incorporating pH- or enzyme-sensitive components can help overcome stromal resistance and achieve deeper tumor infiltration, ultimately improving therapeutic outcomes in patients with lung cancer [197]. The respiratory mucus layer, which lines the trachea and bronchial tree, primarily consists of water, mucins (mainly MUC5AC and MUC5B), and various biomolecules such as DNA, lipids, and proteins. This viscoelastic and negatively charged barrier, formed by glycosylated mucins crosslinked through disulfide bonds and glycan entanglements, serves as a frontline defense against inhaled nanomaterials (NMs). Its dense structure limits the passage of larger particles, with a pore size of approximately 340 ± 70 nm. Furthermore, electrostatic interactions between the negatively charged mucus and positively charged NMs, along with hydrophobic and hydrogen bonding interactions, contribute to the retention of NMs, making the bio-nano interface a significant obstacle for effective pulmonary nanomedicine delivery [197]. These mucus-mediated interactions at the bio-nano interface not only hinder nanoparticle mobility but also exacerbate other physiological barriers such as tumor heterogeneity, airway remodeling, and mucociliary clearance, which together limit consistent and effective pulmonary drug delivery in lung cancer patients.

Although nanoparticle-based inhalation therapies offer promising targeted delivery in lung cancer, multiple barriers hinder clinical translation. Tumor heterogeneity, mucus hypersecretion, and altered airway clearance reduce effective deposition and penetration at tumor sites (Figure 3). Mucociliary clearance [23,146] Alveolar macrophage uptake [162], and enzymatic degradation [169] Further limits nanoparticle residence time and therapeutic efficiency. Additionally, variability in inhalation patterns and respiratory capacity among lung cancer patients complicates the consistent delivery of medication.

## 6. Conclusions

The review underscores the promising potential of nanoparticle-based formulations, such as liposomes and dendrimers, in enhancing lung cancer therapy through targeted drug delivery, improving drug solubility and bioavailability, and minimizing toxicity. Despite this, challenges remain in translating these technologies into clinical use, including optimizing nanoparticle properties, overcoming physiological barriers, and ensuring standardized testing. Additionally, the effectiveness of inhaler devices, such as DPIs, pMDIs, and nebulizers, needs to be improved to optimize drug dispersion and lung deposition. Future research should focus on integrating advanced nanoparticle formulations with better-designed inhaler devices to enhance therapeutic outcomes and ensure the successful clinical application of inhalable nanomedicines in the treatment of lung cancer.

## Figures and Tables

**Table 1:** Comparative Overview of Inhalation Devices for Nano-Formulations in Lung Cancer Therapy adapted from [28].

Device Type	Advantages	Limitations
Dry Powder Inhalers (DPIs)	Propellant-free and environmentally friendly Portable with reusable or disposable options No coordination is needed between inhalation and actuation. High inhalation efficiency	Performance is highly dependent on the patient’s inhalation force and breathing pattern Not suitable for the unconscious, severely ill patients with poor inspiratory flow High formulation and device cost Challenges in maintaining nanostructure during drying and redispersion
Pressurized Metered-Dose Inhalers (pMDIs)	Compact, portable, and easy to use Inexpensive and widely available Capable of rapid drug release	Requires good coordination between inhalation and actuation Low lung deposition efficiency (~10–20%) Contains propellants that may disrupt nanostructures Limited dose capacity per actuation Solvent and shear stress may affect nanoparticle stability
Nebulizers (Jet, Ultrasonic, Vibrating Mesh)	Do not require patient coordination suitable for pediatric, geriatric, and unconscious patients Capable of delivering large doses over extended periods Compatible with a wide range of nano-formulations (solutions and suspensions) Preserve the [11] structural integrity of sensitive nanocarriers	Jet: bulky, noisy, low aerosol output, significant drug residue, poor portability Ultrasonic: not suitable for proteins, suspensions, or heat-sensitive drugs Mesh: expensive and requires maintenance/cleaning -Generally slower administration time than inhalers

**Table 2:** Clinical Trials of Inhaled Nanocarriers for Lung Cancer via different inhalation devices.

Nanoformulations	Type of Cancer	Inhalation Delivery Modality	Ref.
Liposomal 9-Nitrocamptothecin	PC/MC	AeroMist nebulizer	[69]
Liposomal cisplatin	NSCLC/SCLC	Jet nebulizer	[73]
Liposomal cisplatin	MC (osteosarcoma)	Nebulizer	[76]
Gemcitabine	NSCLC	Mesh nebulizer	[77]
Liposomal Doxorubicin	NSCLC/MC	Nebulizer	[78]
* Amikacin Liposome Inhalation Suspension (Arikayce^®^)	Mycobacterium avium Complex (MAC) lung disease (not cancer, but lung application	Nebulizer (Lamira^®^ Nebulizer System)	[75]

NSCLC: non-small cell lung cancer, SCLC: small cell lung cancer, PC: primary cancer in the lungs, MC: metastatic cancer in the lungs. * Although this formulation is not indicated for lung cancer, it has been approved for human inhalation use.

## Data Availability

Not applicable.
